# Expression of ADAM10 and CD58 in Acute and Chronic Lymphocytic Leukemia: Influence of Disease Stage and Chemotherapy 

**DOI:** 10.30699/ijp.2025.2060663.3459

**Published:** 2025-07-01

**Authors:** Ameer Hamid Kadhim, Muslim Idan Mohsin

**Affiliations:** Department of Pathological Analyses, Faculty of Science, University of Kufa, Kufa, Najaf Governorate, Iraq

**Keywords:** CD58, ADAM10, acute lymphoblastic leukemia, chronic lymphocytic leukemia, doxorubicin, vincristine, methotrexate

## Abstract

**Background & Objective:**

CD58 and ADAM10 have been implicated in leukemia progression and chemoresistance; however, their specific roles in acute lymphoblastic leukemia (ALL) and chronic lymphocytic leukemia (CLL), particularly under chemotherapeutic pressure, remain insufficiently characterized. This study aimed to assess the expression of CD58, an immune adhesion molecule, and ADAM10, a metalloproteinase, in ALL and CLL patients undergoing chemotherapy, and to explore their potential involvement in immune evasion, niche-mediated survival, and chemoresistance mechanisms.

**Methods:**

Peripheral blood mononuclear cells (PBMCs) were isolated from 50 patients with ALL, 50 with CLL, and 30 healthy controls. Expression levels of CD58 and ADAM10 were analyzed by quantitative reverse transcription PCR (qRT-PCR) and flow cytometry. Chemotherapy regimens included vincristine (VCR), methotrexate (MTX), and doxorubicin (DOXO).

**Results:**

ADAM10 mRNA expression was significantly upregulated in ALL patients treated with VCR+MTX (p<0.0001) and DOXO (p=0.001), with corresponding protein overexpression observed in both ALL (p<0.0001) and untreated CLL patients (p<0.0001). A significant difference in ADAM10 levels was noted between ALL and CLL cohorts (p=0.001). CD58 mRNA and protein expression were markedly increased in ALL patients receiving VCR+MTX (p<0.0001), whereas untreated CLL patients exhibited no significant alterations.

**Conclusion:**

CD58 and ADAM10 are differentially regulated in ALL under chemotherapy, supporting their roles in immune evasion and microenvironmental survival. The constitutive overexpression of ADAM10 in CLL suggests its involvement in chronic leukemic pathogenesis. These findings highlight CD58 and ADAM10 as potential therapeutic targets for overcoming chemoresistance in lymphoid malignancies.

## Introduction

ADAM10, a transmembrane zinc-dependent metalloprotease, plays a crucial role in acute leukemia (ALL) by regulating leukemia stem cell (LSC) survival and interactions within the bone marrow niche (1, 2). Its function, modulated by Tspan8 and SAP97 (3), involves the shedding of surface proteins like NOTCH1, impacting oncogenic signaling and LSC dormancy (1, 4). Consequently, ADAM10 inhibition disrupts leukemia cell homing, reduces stem cell frequency, and enhances chemosensitivity in preclinical models (1, 2, 5). While ADAM10's direct involvement in chronic lymphocytic leukemia (CLL) is less defined, it is implicated in other B-cell malignancies (6). 

CD58, an immunoglobulin superfamily member overexpressed in precursor-B ALL blasts (7), serves as an MRD marker and modulates immunotherapy responses. Its role in effector-target adhesion during antigen recognition (8). underscores its importance in immune responses, although antigen-independent adhesion mechanisms also exist (9). While its role in CLL requires further investigation, CD58 is relevant in diffuse large B-cell lymphoma (10). Chemotherapeutic agents like Doxorubicin (Doxo), Vincristine (VCR), and Methotrexate (MTX) are integral to ALL treatment (11). The primary objective of this study is to meticulously quantify the messenger RNA (mRNA) expression levels of the *ADAM10* and *CD58* genes within peripheral blood mononuclear cells (PBMCs). This quantification will be conducted across three distinct cohorts: patients diagnosed with acute lymphoblastic leukemia (ALL), patients with chronic lymphocytic leukemia (CLL), and healthy control individuals, utilizing the highly sensitive real-time RT-PCR technique. Concurrently, the study aims to assess the protein expression levels of CD58 and ADAM10 directly on PBMCs from these same patient and control groups through the application of flow cytometry. Ultimately, by achieving these objectives, this research endeavors to investigate and ascertain the potential of ADAM10 and CD58 as valuable biomarkers for the diagnosis, prognosis, or monitoring of lymphocytic leukemia.

## Materials and Methods

### Study Population and Recruitment

The study is scheduled to be conducted from May 1st to September 22nd, 2024, targeting a comprehensive sample size of 130 participants. This cohort will be composed of 50 patients diagnosed with acute lymphoblastic leukemia (ALL), 50 patients with chronic lymphocytic leukemia (CLL), and 30 healthy control individuals. Recruitment for all participants will be exclusively carried out at the Cancer Oncology Department of Al-Forat Al-Awsat Hospital in Najaf, Iraq. A crucial aspect of patient classification will involve considering disease activity, specifically categorizing both ALL and CLL patients based on their current chemotherapy treatment status and the stage of their disease. Chemotherapy regimens relevant to this classification include vincristine (VCR), methotrexate (MTX), and doxorubicin (DOXO), reflecting the specific treatment cycles received. This sub-classification will be meticulously defined and documented during the data collection phase.

Inclusion criteria for ALL/CLL patients necessitate a confirmed diagnosis by a board-certified hematologist at Al-Forat Al-Awsat Hospital, adhering to established diagnostic criteria for leukemia. This diagnosis must be further corroborated by flow cytometric analysis utilizing commercially available IVD antibody kits. Healthy controls will comprise age and sex-matched individuals with no documented history of hematological malignancies or other chronic diseases. Conversely, exclusion criteria will apply to patients with other forms of leukemia or malignancies, as well as individuals currently undergoing chemotherapy or radiation therapy that could potentially confound biomarker expression. For healthy control participants, any pre-existing condition affecting blood cell counts or the immune system will serve as grounds for exclusion.

### Isolation of Peripheral Blood Mononuclear Cells (PBMCs)

Peripheral blood mononuclear cells (PBMCs) were isolated from whole blood using Ficoll density gradient centrifugation, following the manufacturer's protocol (SolarBio, Lot No. P4350) with minor modifications. Briefly, whole blood was diluted 1:1 with sterile phosphate-buffered saline (PBS) and carefully layered over Ficoll-Hypaque at a 2:1 ratio. After centrifugation at 1400×g for 40 minutes at room temperature (brake off), the PBMC layer was harvested from the plasma-Ficoll interface using a sterile Pasteur pipette. The cells were washed twice with PBS (300×g, 10 minutes each) to remove residual Ficoll and contaminants. The resulting cell pellet was resuspended in freezing medium (FCS supplemented with 5% dimethyl sulfoxide), and cell counts were determined using an automated cell counter (Bio-Rad). The PBMC suspension was adjusted to a final concentration of 2×10⁶ cells/mL in freezing medium and cryopreserved in liquid nitrogen for subsequent quantitative polymerase chain reaction (qPCR) analysis (12).

### ADAM10 and CD58 Expression in PBMCs as a Potential Biomarker in Lymphocytic Leukemia: A Real time RT-PCR Study. 

(PBMCs) were obtained from healthy controls, patients with active disease, and patients in remission to establish distinct experimental cohorts. Total RNA was extracted from all samples using the Solarbio Life Science RNA extraction kit, following the manufacturer's protocol. Briefly, cells were centrifuged (200 x g, 5 minutes), lysed, and RNA purified through sequential washes with RPE buffer and two washes with WT buffer. Purified RNA was eluted and stored at -20°C until further processing. Complementary DNA (cDNA) was synthesized via reverse transcription. Quantitative PCR (qPCR) was performed using the Primer Design Precision 2x qPCR SYBR Green Master Mix on an Applied Biosystems 7900HT Fast Real-Time PCR System. Pre-designed, validated primer/probe sets (Macrogen, South Korea) targeting the GAPDH, CD58 and ADAM10 gene as in table 2 below. Amplification was carried out for 40 cycles. Relative GAPDH, CD58 and ADAM10 gene expression was determined using the comparative cycle threshold (Ct) method (13), normalizing to GAPDH expression. All of the primers listed below were designed as part of this specific study and their sequences were obtained from the NCBI BLAST tool https://blast.ncbi.nlm.nih.gov/Blast.cgi.

### ADAM10 and CD58Expression in PBMCs as a Potential Biomarker in Lymphocytic Leukemia: A Flow Cytometric Study.

CD58 and ADAM10 expression on peripheral blood mononuclear cells (PBMCs) from patients with acute lymphoblastic leukemia (ALL), chronic lymphocytic leukemia (CLL), and healthy controls was assessed by flow cytometry at protein level. CD58 and ADAM10 is predominantly cell surface proteins, making flow cytometry a suitable method for their detection. Briefly, PBMCs were harvested and washed twice with ice-cold wash buffer (phosphate-buffered saline containing 1% bovine serum albumin and 0.1% sodium azide). Washed cells were resuspended at a concentration of 0.5 x 10⁶ cells per test. A 50 µL aliquot of conjugated mouse anti- CD58 and ADAM10 antibody (50 µg/mL) was added to each cell suspension, and the mixture was incubated for 45 minutes on ice. Following incubation, cells were washed twice (400 x g for 5 minutes) with wash buffer. Subsequently, cell pellets were resuspended in 300 µL of wash buffer and immediately analyzed using a BD FACSCanto II flow cytometer. Ten thousand live events were acquired for each sample. Data analysis was performed using FlowJo software. Cell populations were gated based on forward scatter (FSC) and side scatter (SSC) parameters to define size and granularity, respectively. 

### Statistical Analyses

For statistical analysis and creating graphs, we used GraphPad Prism version 10. To compare the different experimental groups, we used a statistical test called one-way ANOVA. We chose this test because it's suitable for our experimental setup. Since we had multiple groups, we needed to do extra tests after the ANOVA. These "post-hoc tests" (either Tukey's or Bonferroni) helped us pinpoint exactly which groups were significantly different from each other while accounting for the fact that we were doing multiple comparisons. All our data are presented as the average value (mean) plus or minus the standard error of the mean SEM. Before running these tests, we confirmed that our data followed a normal distribution and met the criteria for parametric statistical tests. Therefore, the specific post-hoc test used was either Tukey's or Bonferroni, depending on the specific comparisons being made.

**Table 1:** List of Human Primers and PCR Product Sizes

Name	Human primers	PCR product
CD58 F	ATCCCAAGCAGCGGTCATTC	165
CD58 R	GCTGTTGTCTTCATCTTCTGTTACC	165
ADAM10 F	ACCAGATGACTGGTGTAGAGGA	197
ADAM10 R	AGCAGTTGTATTGATCTGGGCA	197
GAPDH F	GTCGGAGTCAACGGATTTGG	165
GAPDH R	GACGGTGCCATGGAATTTGC	165

## Results

**Chemotherapy-Specific ADAM10 Modulation in Acute vs. Chronic Leukemia**

**Figure 1:**
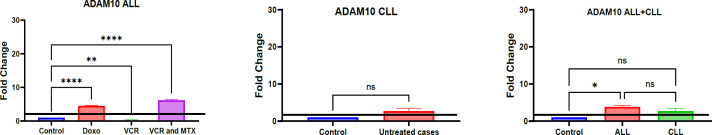
the changes in ADAM10 mRNA expression in patients ALL and CLL

**Fig. 3:**
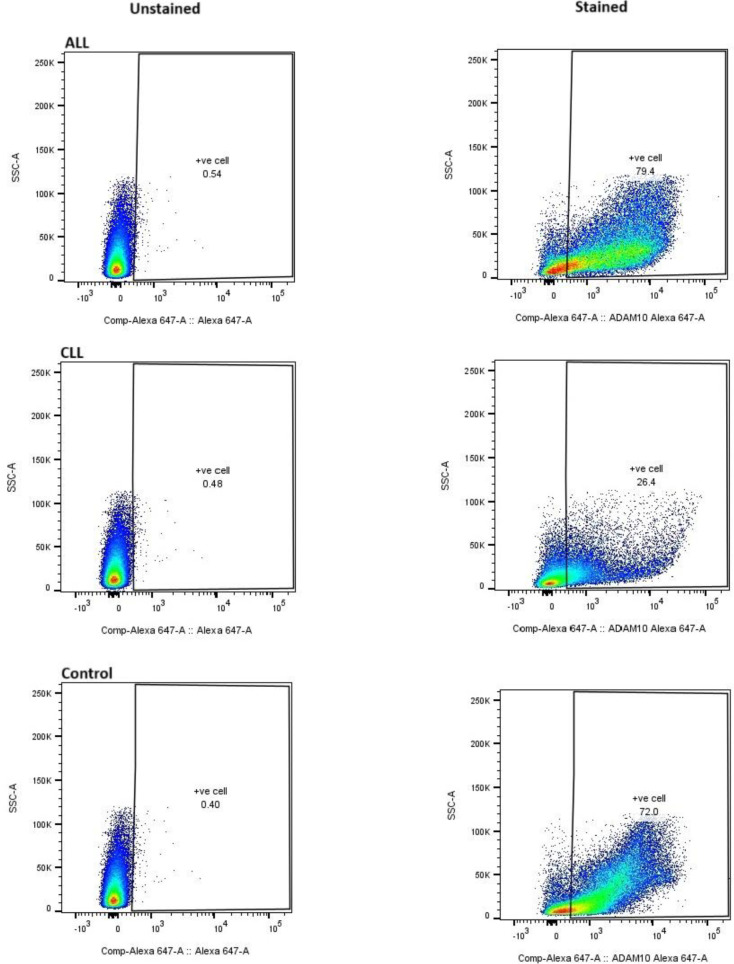
Flow Cytometry Analysis of ADAM10 Expression in Healthy Controls, Acute Lymphoblastic Leukemia (ALL), and Chronic Lymphocytic Leukemia (CLL) Patients. PBMCs were stained with a fluorescently-conjugated antibody specific for ADAM10. Unstained PBMCs were used as a negative control to determine background fluorescence. Data were acquired using a BD Flow Cytometer device and analyzed using Flow JO software. Anti- ADAM10 antibody (K011477M) is shifted with Alexa 647 detector as in above.

This section, illustrated by Figure 1 for mRNA levels and Figure 2 for protein levels, aimed to comprehensively evaluate the regulation of ADAM10 expression in both acute lymphoblastic leukemia (ALL) and chronic lymphocytic leukemia (CLL) cases, specifically examining the impact of chemotherapeutic treatments (vincristine, methotrexate, and doxorubicin) on ALL samples. Analysis of ADAM10 mRNA expression revealed a notable dependence on the type of chemotherapy administered in ALL cases. Specifically, a statistically significant upregulation of ADAM10 mRNA was observed in the ALL group treated with a combination of vincristine (VCR) and methotrexate (MTX) (P=0.0001,∗∗∗∗) and in the group treated with doxorubicin (DOXO) (P=0.001,∗∗∗). Conversely, treatment with VCR alone did not result in a statistically significant alteration in ADAM10 mRNA expression. In untreated CLL cases, ADAM10 mRNA expression also showed no statistically significant change compared to controls. Furthermore, no significant differences in ADAM10 mRNA levels were detected between the treated ALL groups and the control cases, or between the ALL and CLL groups overall. Parallel analysis of ADAM10 protein expression, as presented in Figure 2, demonstrated a general trend of increased ADAM10 protein levels in both ALL and CLL cases. Within the treated ALL subgroups, significant upregulation of ADAM10 protein was observed in the VCR and MTX combination group (P=0.01,∗∗) and the DOXO-treated group (P=0.0001,∗∗∗∗), mirroring the mRNA findings. The VCR-only group again showed a non-significant change in protein expression. Interestingly, ADAM10 protein expression was also significantly increased in untreated CLL cases (P=0.0001,∗∗∗∗). Moreover, a statistically significant difference in ADAM10 protein levels was found between ALL and CLL cases (P=0.001,∗∗∗). The specificity of the ADAM10 antibody used in these analyses is confirmed by the data presented in Figure 3, which compares staining in healthy controls, ALL, and CLL cases against unstained controls, demonstrating specific binding.

Expression of ADAM10 in healthy cases and non-healthy cases was calculated using the 2-ΔΔCt method following estimation of the housekeeping gene GAPDH. ADAM10 showed change in PBMCs. The significance of differences has been tested by one-way ANOVA, where *** p<0.001, and **** p<0.0001 is significant, and ns is non-significant.

**Fie 2:**
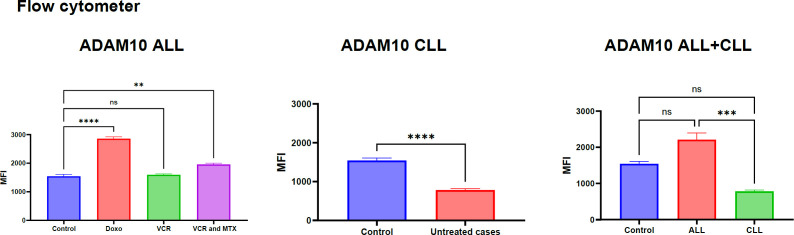
The changes in the ADAM10 at protein level in response to ALL and CLL at different types of chemotherapies.

Expression of ADAM10 in healthy cases and non-healthy cases was calculated using the MFI method. ADAM10 showed change in PBMCs. The significance of differences has been tested by one-way ANOVA, where ** p<0.01, *** p<0.001, and **** p<0.0001 is significant, and ns is non-significant. The data are the means of 130 samples from three separate experiments with duplicates.

**Chemotherapy-Driven CD58 Overexpression in ALL: A Contrast with Untreated CLL**

**Fig. 4:**
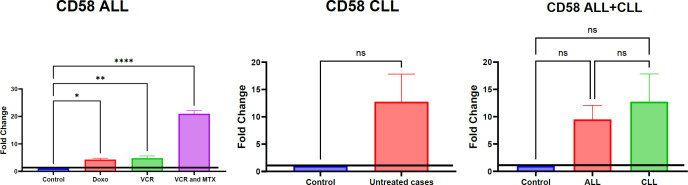
the changes in CD58 mRNA expression has been measured by qPCR in patients.

**Fig. 5:**
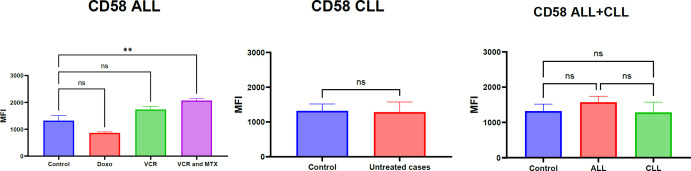
The changes in the CD58 at protein level in response to ALL and CLL at different types of chemotherapies. Expression of CD58 in healthy cases and non-healthy cases was calculated using the MFI method. CD58 showed change in PBMCs. The significance of differences has been tested by one-way ANOVA, where ** p<0.01 is significant, and ns is non-significant. The data are the means of 130 samples from three separate experiments with duplicates.

This section, illustrated by Figure 4 for mRNA and protein levels Figure 5, aimed to investigate the regulation of CD58 expression in both treated acute lymphoblastic leukemia (ALL) and untreated chronic lymphocytic leukemia (CLL) cases, specifically examining the impact of vincristine, methotrexate, and doxorubicin chemotherapy on ALL samples.

Analysis of CD58 mRNA expression ALL cases revealed a significant overall increase. Notably, the effect of chemotherapy on CD58 mRNA levels varied depending on the specific regimen. A statistically significant upregulation was observed in the ALL group treated with a combination of vincristine (VCR) and methotrexate (MTX) (P=0.0001,∗∗∗∗), the VCR-only group (P=0.01,∗∗), and the doxorubicin (DOXO) group (P=0.01,∗). In contrast, CD58 mRNA expression in untreated CLL cases remained statistically non-significant compared to controls. Furthermore, no significant differences in CD58 mRNA levels were detected between the treated ALL groups and the control cases, nor between the ALL and CLL groups overall.

**Fig 6:**
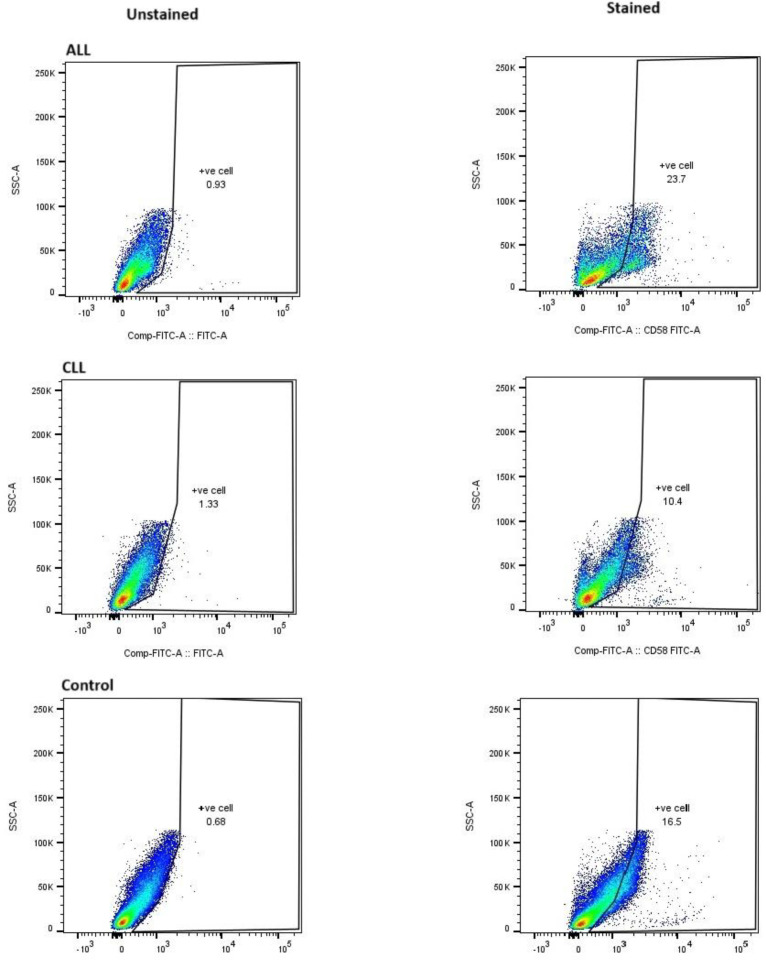
Flow Cytometry Analysis of CD58 Expression in Healthy Controls, Acute Lymphoblastic Leukemia (ALL), and Chronic Lymphocytic Leukemia (CLL) Patients.

Parallel investigation of CD58 protein expression ALL cases, as depicted in Figure 3b, also demonstrated a significant overall increase in ALL. Specifically, the combination treatment of VCR and MTX resulted in a statistically significant upregulation of CD58 protein (P=0.01,∗∗). However, unlike the mRNA results, the VCR-only and DOXO-treated groups showed non-significant changes in CD58 protein expression. Similar to the mRNA findings, CD58 protein expression in untreated CLL cases remained statistically non-significant, and no significant differences were observed between the treated ALL groups and control cases, or between the ALL and CLL groups. The specificity of the CD58 antibody employed in these analyses is confirmed by the data presented in Figure 6, which demonstrates specific binding in healthy controls, ALL, and CLL cases compared to unstained controls.

Expression of CD58 in healthy cases and non-healthy cases was calculated using the 2-ΔΔCt method following estimation of the housekeeping gene GAPDH. CD58 showed change in PBMCs. The significance of differences has been tested by one-way ANOVA, where * p<0.1, ** p<0.01, and **** p<0.0001 is significant, and ns is non-significant.

PBMCs were stained with a fluorescently-conjugated antibody specific for CD9. Unstained PBMCs were used as a negative control to determine background fluorescence. Data were acquired using a BD Flow Cytometer device and analyzed using Flow JO software. Anti- CD58 antibody (K010825M) is shifted with FITC detector as in above.

## Discussion

In this study, we investigated the potential roles of ADAM10 and CD58 proteins in acute lymphoblastic leukemia (ALL) and chronic lymphocytic leukemia (CLL), both in their treated and untreated states, by comparing their expression levels to a healthy control group. Furthermore, we specifically examined how different chemotherapeutic drugs-doxorubicin, methotrexate, and vincristine – affect the expression of these proteins in ALL.

Our analysis of ADAM10 expression, detailed in Figures 1a, 1b, and 3, revealed a statistically significant upregulation at both the mRNA and protein levels in Acute Lymphoblastic Leukemia (ALL) cases following treatment with a combination of vincristine (VCR) and methotrexate (MTX), as well as with doxorubicin (DOXO). Interestingly, VCR monotherapy did not elicit a significant alteration in ADAM10 expression. In contrast, ADAM10 expression remained non-significant in untreated Chronic Lymphocytic Leukemia (CLL) samples. Notably, while ADAM10 protein levels demonstrated an overall increase in CLL compared to healthy controls and a significant difference when compared to ALL, corresponding mRNA levels did not exhibit these distinctions. These findings resonate with the established involvement of ADAM10 in hematological malignancies (14).

The observed upregulation of ADAM10 in ALL upon exposure to combination chemotherapy (VCR+MTX) and DOXO suggests a potential drug-induced response with significant ramifications for treatment efficacy and disease progression. ADAM10, a key sheddase of numerous transmembrane proteins, including receptors and ligands critical for cell signaling, may contribute to the shedding of therapeutic targets on ALL cells following chemotherapy (14, 15).This shedding could represent a novel mechanism of drug resistance or altered drug sensitivity in these patients. Furthermore, the modulation of the tumor microenvironment through the shedding of specific molecules by upregulated ADAM10 could influence crucial processes such as cell adhesion, migration, and immune evasion. In line with this, the release of soluble ectodomains due to heightened ADAM10 activity might themselves act as signaling molecules, potentially fostering survival and proliferation signals in leukemia cells. Our findings align with recent studies highlighting the critical role of ADAM10 in acute leukemia and its potential as a therapeutic target (16). Indeed, preclinical evidence suggests that ADAM10 inhibition can augment the efficacy of conventional chemotherapy in leukemia models (17). Other researchers have also confirmed our findings, ADAM10 plays a significant role in T-cell acute lymphoblastic leukemia (T-ALL) by contributing to the aberrant activation of oncogenic NOTCH1(18, 19). Research indicates that both ADAM10 and ADAM17 metalloproteases are involved in the S2 processing of NOTCH1 in T-ALL, leading to its activation. This is further supported by studies showing that the ADAM10 inhibitor GI254023X can inhibit Jurkat cell proliferation, induce apoptosis, and modulate NOTCH1 expression (down-regulating Cleaved Notch1 and up-regulating total Notch1), providing strong evidence for ADAM10's involvement in T-ALL pathogenesis (20). The discordant expression pattern of ADAM10 between mRNA and protein levels in CLL, characterized by elevated protein but not mRNA, suggests the presence of post-transcriptional regulatory mechanisms in this specific leukemia subtype (21). This could involve enhanced mRNA translation, increased protein stability, or distinct regulatory pathways governing ADAM10 expression in CLL compared to ALL. Moreover, the significant difference in ADAM10 protein levels between ALL and CLL underscores a potentially divergent role for this protease in the pathogenesis or progression of these two distinct hematological malignancies. Studies have identified ADAM10 as the primary sheddase of CD23, a molecule of relevance in CLL, and have implicated its role in Notch signaling, a pathway frequently dysregulated in various cancers, including leukemia (14). Furthermore, research in other hematological malignancies, such as Hodgkin lymphoma, has demonstrated the expression of ADAM10 and the potential for its inhibition to exert anti-tumor effects and enhance the activity of other therapeutic agents (22, 23).A deeper understanding of these processes may pave the way for the development of targeted therapeutic strategies aimed at modulating ADAM10 activity in these hematological malignancies.

Our analysis of CD58 expression, as illustrated in Figures 3a and 3b, revealed a significant overall increase at both the mRNA and protein levels in Acute Lymphoblastic Leukemia (ALL) cases. Notably, the combination of vincristine (VCR) and methotrexate (MTX) significantly upregulated both CD58 mRNA and protein. Interestingly, while VCR alone and doxorubicin (DOXO) also led to significant increases in CD58 mRNA, these changes were not reflected at the protein level. Consistent with ADAM10 mRNA findings, CD58 expression in untreated Chronic Lymphocytic Leukemia (CLL) cases remained statistically non-significant, and no significant differences were observed between treated ALL groups and controls, or between ALL and CLL groups. The specificity of the CD58 antibody was confirmed in Figure 4. The major finding in this study was the association between CD58 expression and prognosis of ALL. These findings align with existing literature highlighting the involvement of CD58 in ALL and CLL (7, 24). These findings are consistent with existing research that highlights CD58 involvement in both Acute Lymphoblastic Leukemia (ALL) and Chronic Lymphocytic Leukemia (CLL). CD58, also known as Lymphocyte Function-Associated Antigen-3 (LFA-3), is a key molecule. It acts as a ligand for CD2, which is found on T cells and Natural Killer (NK) cells. This interaction is crucial for cell adhesion and for providing the costimulatory signals necessary for effective immune responses (25). In the context of ALL, increased CD58 expression on leukemic blasts could significantly impact their interaction with immune effector cells, potentially influencing immune surveillance and the ability of the immune system to eliminate malignant cells (26, 27). The synergistic effect of the VCR and MTX combination on both CD58 mRNA and protein levels in ALL warrants further investigation to elucidate the underlying signaling pathways (28). The intriguing discordance between CD58 mRNA and protein levels observed with VCR and DOXO monotherapy strongly suggests the involvement of post-transcriptional regulatory mechanisms, including miRNA-mediated regulation (29), RNA-binding protein involvement (30), or protein degradation pathways (31). The non-significant CD58 expression in untreated CLL cases, similar to our findings with ADAM10 mRNA, suggests a potentially distinct regulatory landscape for CD58 in CLL compared to ALL (32). The lack of significant differences in CD58 expression between treated ALL groups and controls, as well as between ALL and CLL groups when considering all treated ALL cases together, might reflect patient heterogeneity or the dynamic nature of CD58 regulation in response to chemotherapy.

Considering the observed upregulation of both ADAM10 and CD58 in ALL following specific chemotherapy regimens, particularly the VCR+MTX combination, a potential functional link warrants exploration. ADAM10 is known to shed the extracellular domain of various cell surface proteins. While CD58 is not a well-established direct substrate of ADAM10, the parallel upregulation of these two molecules in response to chemotherapy raises the possibility of an indirect relationship. For instance, ADAM10-mediated shedding of another transmembrane protein could influence signaling pathways that subsequently lead to increased CD58 expression. Alternatively, the cellular stress induced by chemotherapy might independently trigger the upregulation of both ADAM10 and CD58 through distinct but potentially interconnected pathways. Further investigation into the downstream effects of ADAM10 upregulation in this context, including secretome analysis and examination of cell surface protein shedding, could provide insights into whether CD58 or other immune-related molecules are potential targets of ADAM10 in chemotherapy-treated ALL cells. Understanding the interplay between ADAM10 and CD58 regulation in response to treatment may reveal novel mechanisms of chemoresistance or immune modulation in ALL.

## Conclusion

Our findings indicate that the chemotherapeutic regimens employed differentially regulate the expression of ADAM10 and CD58 in ALL.The combination of VCR and MTX consistently induced significant upregulation of both targets at both mRNA and protein levels. Furthermore, while ADAM10 protein showed elevated basal levels in CLL compared to ALL, CD58 expression remained largely unchanged in untreated CLL cases.
